# Seroprevalence of SARS-CoV-2 in rural communities of Burkina faso (West Africa) assessed through blood-fed mosquitoes

**DOI:** 10.1038/s41598-026-46133-5

**Published:** 2026-04-02

**Authors:** Raïssa Muriel de Souza, Etienne Bilgo, Edounou Jacques Gnambani, Djibril Samaké, Abdoul Azize Millogo, Maurice Konkobo, Dramane Kania, Adama Dao, Tovi Lehmann, Abdoulaye Diabate

**Affiliations:** 1 Centre d’excellence Africain en Innovation Biotechnologiques pour le Contrôle des maladies à Transmission Vectorielle (CEA-ITECH/ MTV), Université Nazi Boni, Bobo Dioulasso, Burkina Faso; 2 Institut National de Santé Publique / Centre Muraz, Bobo Dioulasso, Burkina Faso; 3 Institut de Recherche en Sciences de la Santé, BP 545, Direction Régionale de l’Ouest, Bobo- Dioulasso, Burkina Faso; 4 Malaria Research and Training Center (MRTC), Faculty of Medicine, Pharmacy and Odonto- Stomatology, University of Sciences, Techniques and Technologies, Bamako, Mali; 5 Institut des Sciences Des Sociétés (INSS), Ouagadougou, Burkina Faso; 6 Laboratory of Malaria and Vector Research, NIAID, NIH, Rockville, MD USA

**Keywords:** SARS-CoV-2, Seroprevalence, Mosquito blood-meal, Rural Communities, Burkina Faso, Diseases, Health care, Medical research, Microbiology

## Abstract

Since the emergence of the COVID-19 pandemic, several surveillance measures have been implemented in African cities, including the continuous collection and analysis of epidemiological data to identify suspected or confirmed cases. Rural regions, where over half of Africa’s population resides, often face a scarcity of quality health care infrastructure and diagnostic services, hindering effective disease surveillance and potentially accelerating disease transmission. Our study aimed to assess COVID-19 seroprevalence and spread in rural Burkina Faso using an innovative noninvasive sampling method based on naturally blood-feeding mosquitoes as a proxy for human sampling. This cross-sectional study was conducted from October to November 2021 in eleven rural localities located along the RN1 road in Burkina Faso, including six villages near Bobo-Dioulasso (Bereba, Bombi, Dimikuy, Intiedougou, Koumbia, and Nahirindon) and five villages near Ouagadougou (Ouezindougou, Saria, Sissene, Tampelga, and Tathyou). Samples consisted of 690 blood-fed mosquitoes collected by aspiration from 299 human dwellings. The predominant vector species were *Anopheles funestus* and *Anopheles gambiae*. ELISA tests were performed to detect human anti-spike protein and anti-RBD domain antibodies to SARS-CoV-2. Overall, SARS-CoV-2 seroprevalence detected in mosquitoes was 31.16% (215/690). Seroprevalence was higher in rural areas near Bobo-Dioulasso, reaching 35.14% (130/370), compared with 26.56% (85/320) in areas near Ouagadougou. In addition to providing evidence of SARS-CoV-2 circulation during an active phase of the pandemic, this study evaluated the feasibility of using naturally fed mosquitoes as a complementary surveillance tool for monitoring SARS-CoV-2 seroprevalence in rural Burkina Faso. However, due to missing data on the time each mosquito took the blood meal, its source, and the proportion of mosquitoes feeding on the same individual hosts, this information serves as a crude minimum infection rate in these populations and additional sampling, and assays are needed to accurately extend these results to determine exact seroprevalence in these communities.

**Supplementary Information:**

The online version contains supplementary material available at 10.1038/s41598-026-46133-5.

## Background

Emerging infectious diseases (EIDs) pose a serious threat to the global health system and cause millions of deaths and casualties every year^1^. The emergence of the SARS-CoV-2 responsible for severe acute respiratory syndrome, or COVID-19, in Wuhan in December 2019 is an especially relevant example^2^. COVID-19 has spread rapidly around the world and by August 2023 has caused more than 769 million SARS-CoV-2 infections and 11.7 million deaths^3^. In Burkina Faso, national surveillance reported 22,056 confirmed cases and 396 deaths as of August 2023^4^. These surveillance data are primarily derived from suspected cases and targeted screening of travelers reported through healthcare and control facilities concentrated in urban areas, a limitation that has been widely documented in African countries with comparable surveillance systems^5^. However, sero-epidemiological studies indicate substantially higher levels of SARS-CoV-2 exposure in major urban centers, with estimated seroprevalence reaching 87% in Bobo-Dioulasso and 92% in Ouagadougou in early 2022^6^.

In Africa, a great deal of attention and action has been devoted to implementing control measures .e.g., hand-washing, mostly in urban areas. In rural areas, however, where more than 50% of the population lives^7^, access to health services is limited and COVID-19 diagnostics are seldom available. The lack of systematic monitoring in rural areas contributes to the spread of the disease going undetected. Effective surveillance in these settings is therefore essential to detect emerging pathogens such as SARS-CoV-2, monitor changes in prevalence, and guide public health interventions^8^.

In Burkina Faso, COVID-19 surveillance has mainly been integrated into routine respiratory disease surveillance systems, relying on the reporting of clinically diagnosed cases, COVID-19-attributed mortality, and screening using PCR and/or antigen-based tests^9^. Despite efforts to improve surveillance coverage in rural areas, developing countries such as Burkina Faso continue to face major challenges, including shortages of trained health personnel, limited healthcare infrastructure, insufficient financial resources to support large-scale surveillance, and the geographical isolation of many rural communities^10^. Additionally, it is estimated that less than 20% of patients experiencing fever visit health facilities^8–10^ which also limits the representativeness of facility-based surveillance data.

These limitations highlight a need for alternative, less complex, lower-cost, and field-adapted surveillance strategies that can be deployed at scale in rural settings to complement conventional approaches. One such approach is xenosurveillance, a mosquito-based method that exploits the blood meals of hematophagous arthropods to sample vertebrate blood and monitor circulating pathogens or host immune responses^8,11–13^, Originally proposed more than half a century ago, xenosurveillance has gained renewed interest as a tool for examining the human–pathogen landscape in settings where direct human sampling is logistically challenging or ethically constrained.

Mosquitoes are well known vectors of numerous parasitic and viral diseases. Although SARS-CoV-2 cannot replicate in mosquitoes, even when they feed on viremic hosts^13^. mosquito blood meals represent a valuable source of vertebrate-derived biological material. These blood meals can be exploited to assess antibody seroprevalence in the hosts on which mosquitoes have fed^14^. Previous studies have demonstrated the successful detection of antibodies to dengue virus and *Japanese encephalitis* virus in mosquito blood meals^14,15^. Recently, human antibodies to pathogens not transmitted by mosquitoes, including *Toxoplasma gondii* and SARS-CoV-2, have also been detected using this approach^16,17^. In addition, recent work has shown that mosquito blood meals can be used to characterize vertebrate diversity, host preference, and pathogen exposure in both human and wildlife populations^18^, further highlighting the broad applicability of xenosurveillance beyond classical vector-borne diseases.

Xenosurveillance using blood-fed mosquitoes is non-invasive, logistically simpler, and less costly than traditional serological surveys. Sample storage is minimal and inexpensive, and sample quality does not appear to be compromised by mosquito collection, as no significant differences have been observed between xenosurveillance-derived samples and conventional fingerprint-based sampling methods^12^.

Despite evidence of widespread SARS-CoV-2 circulation in urban areas, epidemiological data from rural communities in Burkina Faso remain extremely limited due to constraints in healthcare access and surveillance capacity. This gap hampers an accurate understanding of SARS-CoV-2 seroprevalence and transmission dynamics in these populations.

The objectives of this study were therefore twofold: to estimate SARS-CoV-2 seroprevalence in rural communities of Burkina Faso using blood-fed mosquitoes as a proxy for direct human sampling, and to evaluate the feasibility of modified xenosurveillance as a complementary surveillance tool for monitoring SARS-CoV-2 exposure in rural settings. To achieve these objectives, blood-fed mosquitoes were collected and analyzed following the approach described by Krajacich et al.^17^, using mosquitoes as stand-ins for conventional human serological sampling.

## Methods

### Study areas

**Fig. 1:**
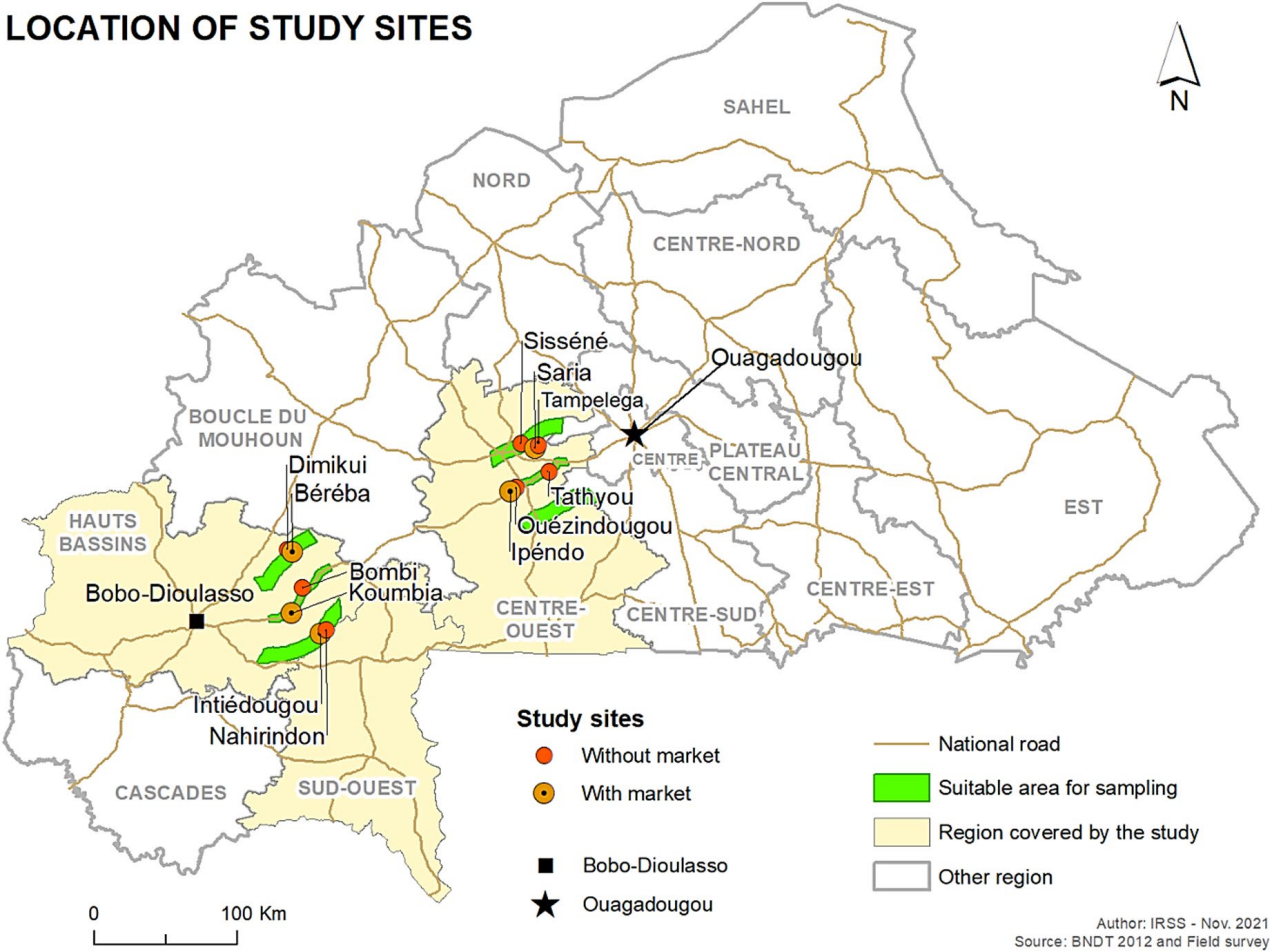
Map of mosquitoes sampling sites. The map was generated using ArcGIS Pro (ESRI), version 2.0 (available at: https://pro.arcgis.com/fr/pro-app/latest/get-started/download-arcgis-pro.htm#GUID-0717B61C-92A2-4B87-9C27-53FBC0F481AA ).

The mosquito sampling sites were selected based on their proximity to the two largest cities in the country where most COVID-19 cases were reported. Locations at 50 km from Bobo-Dioulasso in the Hauts-Bassins region and 50 km from Ouagadougou in the Centre region were selected based on their proximity to the country’s two largest cities, where most cases of COVID-19 have been reported. (Fig. 1). In the Hauts-Bassins region, six villages were selected for mosquito collection. The villages selected were Koumbia (11°23’7074 N -3°69’5902 W); Bombi (11°39’6635 N -3°62’8572 W); Béreba (11°62’7374 N -3°68’8827 W); Dimikuy (11°64’172 N -3°72’396 W); Nahirindon (11°13’6177 N -3°47’6909 W) and Intiédougou (11°10’881 N -3°51’1892 W). Five villages have been selected in Centre region: Ouezindougou (12°03’3907 N -2°27’8885 W); Tathyou or Nabadogo (12°13’3262 N -2°07’1478 W); Saria (12°27’9528 N -2°15’6724 W); Tampelega (12°29’8273 N -2°13’8029 W) and Siséné (12°30’5744 N -22°44’927 W) were selected.

Each village was divided into four geographical zones according to the points of the compass. In each zone, 10 houses were selected at random from different compounds. (i.e. a total of 40 houses/village). In each household mosquitoes were collected in one occupied bedroom. Initially, ELISA tests were performed at the Malaria Research and Training Centre (in Bamako, Mali (*N* = 400 mosquitoes), and at the Centre MURAZ, Bobo Dioulasso, Burkina Faso (*N* = 290 mosquitoes).

### Collecting and storing gorged mosquitoes

Wild mosquitoes were collected alive from October to November 2021 using manual mouth aspirators between 07:00 and 10:00 a.m. Collectors entered houses after obtaining permission from the owner or a responsible adult and aspirated all visible mosquitoes indoors into labeled cups. Indoor collections were intentionally prioritized because freshly blood-fed mosquitoes, particularly endophilic species, are more likely to rest indoors for several hours after feeding, thereby increasing the probability of capturing recent blood meals suitable for xenosurveillance analyses. Following collection, mosquitoes were examined under a binocular microscope, and only swollen, freshly blood-fed individuals were retained for further analysis. Blood-fed mosquitoes were placed individually in tubes containing silica gel and stored at − 20 °C until laboratory testing.

### Detection of human anti-SARS-CoV-2 antibodies in mosquito blood meals

he detection of human anti–SARS-CoV-2 antibodies from mosquito blood meals was performed following the protocol described by Sagara et al.^19^, with minor adaptations previously validated for West African populations. Each blood-fed mosquito abdomen was dissected and homogenized individually in 150 µl of 1× phosphate-buffered saline (PBS), followed by centrifugation at 13,000 rpm for 10 min. The resulting supernatant was used for serological analyses. Enzyme-linked immunosorbent assays (ELISA) were conducted using full-length SARS-CoV-2 spike protein and receptor-binding domain (RBD) antigens, as previously described^19^.

Negative controls consisted of abdomens from laboratory-reared female mosquitoes that had never taken a blood meal, while the monoclonal antibody CR3022 served as a positive control. Seropositivity thresholds were defined according to Sagara et al. (2021), with cut-off values of 0.306 IU/µl for RBD and 0.674 IU/µl for the spike antigen. Optical densities were read at 450 nm with reference at 630 nm using a BK-EL10A microplate reader (Biobase, China). Samples with absorbance values above the established cut-offs for both antigens were considered positive.

### Data analysis

A mosquito was defined as positive when both ELISA assays (spike and RBD) yielded absorbance values above the defined thresholds^19^. Seroprevalence was calculated as the proportion of positive mosquitoes among the total number of blood-fed mosquitoes tested. Differences in mosquito-based seropositivity among mosquito species were assessed using Pearson’s chi-square test at a 5% significance level. This approach was used to evaluate whether the suitability of xenosurveillance for antibody detection differed according to mosquito species. Statistical analyses were performed using Stata version 16 (StataCorp, USA).

### Ethical considerations

Ethical approval for this study was obtained from the Institutional Ethics Committee of the Institut de Recherche en Sciences de la Santé (IRSS), Burkina Faso. Permission to enter households was obtained from household heads or responsible adults prior to mosquito collection. No direct human sampling was performed.

## Results

The serological thresholds used in this study were based on pre-pandemic mosquito blood meal samples, as established by Krajacich et al.^17^, which served as negative controls and baseline reference values.

ELISA tests were performed exclusively on blood-fed mosquitoes collected indoors, resulting in a total of 690 gorged mosquitoes sampled from 299 households. Of these, 370 mosquitoes were collected from six localities near Bobo-Dioulasso, and 320 mosquitoes from five localities near the Ouagadougou region from October to November 2021.

Four mosquito species were identified among the collected samples: Anopheles gambiae (*N* = 462), Anopheles funestus (*N* = 158), Anopheles rufipes (*N* = 34), and Culex quinquefasciatus (*N* = 36) (Table 1).

**Table 1:** Distribution of gorged mosquitoes collected in different localities according to species.

Locality	Species				*N*( mosquitoes)/ *N*(house)
	C quinquefasciatus	An funestus	An gambiae	An rufipes	
***Area near Bobo Dioulasso***					
Bereba	6	**-**	74	1	**81/33**
Bombi	4	**-**	34	1	**39/25**
Dimikuy	10	**-**	31	**-**	**41/16**
Intiedougou	2	**-**	89	**-**	**91/31**
Koumbia	4	**-**	53	**-**	**57/29**
Nahirindon	3	5	52	1	**61/31**
Total	***29***	***5***	***333***	***3***	***370/165***
***Area near Ouagadougou***					
Ouezindougou	2	85	25	13	**125/34**
Saria	2	17	28	1	**48/18**
Sissene	2	15	32	8	**57/27**
Tampelga	**-**	36	10	8	**54/31**
Tathyou	1	-	34	1	**36/24**
**Total**	**7**	**153**	**129**	**31**	***320/134***
**TOTAL**	**36**	**158**	**462**	**34**	**690/299**

The overall mosquito-based seroprevalence was **31.16% (95% CI: 27.68–34.64).** Seroprevalence was higher in localities near Bobo-Dioulasso **(35.14%**,** 95% CI: 30.27–40.00**) compared with those near Ouagadougou **(26.56%**,** 95% CI: 21.87–30.56)**.

At the village level, the highest mosquito-based seroprevalence was observed in Nahirindon (45.90%, *N* = 61) and Intiedougou (45.06%, *N* = 91), whereas no seropositive mosquitoes were detected in Saria (0%) (Table 2).

Household-level seroprevalence was defined as the proportion of households in which at least one blood-fed mosquito tested positive for SARS-CoV-2 antibodies.

**Fig. 2:**
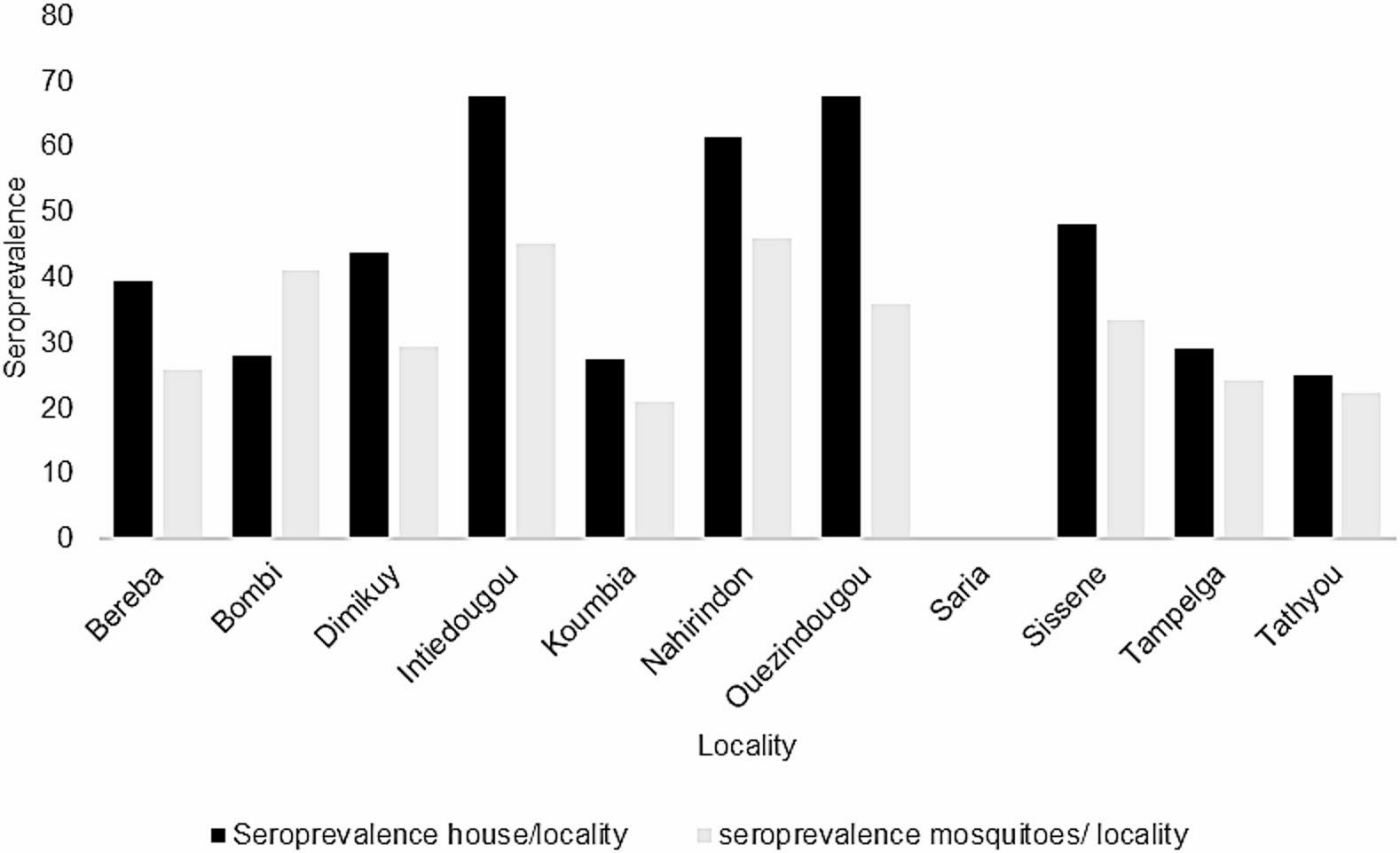
comparison between seroprevalence at the mosquito and the house levels across locality.

Overall, seroprevalence estimates based on households were higher than those based on individual mosquitoes, An exception was observed in Bombi, where mosquito-based seroprevalence exceeded household-based estimates **(**Fig. 2**)**.

**Table 2:** Seroprevalence of COVID-19 in rural areas of Burkina Faso between October and November 2021 using enzyme-linked immunosorbent assay on naturally fed mosquitoes.

Locality	Mosquitoes	House	Total
	% (IC 95%)	% (IC 95%)	*N*( mosquitoes)/ *N*(house)
Area near Bobo Dioulasso			
Bereba	25.93 (17.28–35.80)	39.39 (24.24–57.57)	**81/33**
Bombi	41.03 (25.64–56.41)	28 (12–48)	**39/25**
Dimikuy	26.27 (17.07–43.90)	43.75 (18.75–68.75)	**41/16**
Intiedougou	45.06 (35.16–54.94)	67.74 (51.61–83.87)	**91/31**
Koumbia	21.06 (10.52–31.58)	27.58 (13.79–44.82)	**57/29**
Nahirindon	45.90 (32.79–59.01)	61.29 (45.16–77.41)	**61/31**
*Total*	***35.14 (30.27-40*** **)**	***45.45 (38.5–53.8*** **)**	***370/165***
***Area near Ouagadougou***			
Ouezindougou	36 (28–45)	67.64(52.94–82.35)	**125/34**
Saria	0 (0–0)	0 (0–0)	**48/18**
Sissene	33.33 (21.05–45.61)	48.14 (29.62–66.66)	**57/27**
Tampelga	24.07 (13-35.18)	29.03 (12.90-45.16)	**54/31**
Tathyou	22.22 (9-36.11)	25.00 (8.33–41.66)	**36/24**
*Total*	***26.56 (21.87–30.56)***	***38.06 (30.06–46.57*** **)**	***320/134***
***TOTAL***	**31.16 (27.68–34.64)**	**42.14** ***(36.59–47.88*** **)**	**690/299**

**Fig. 3:**
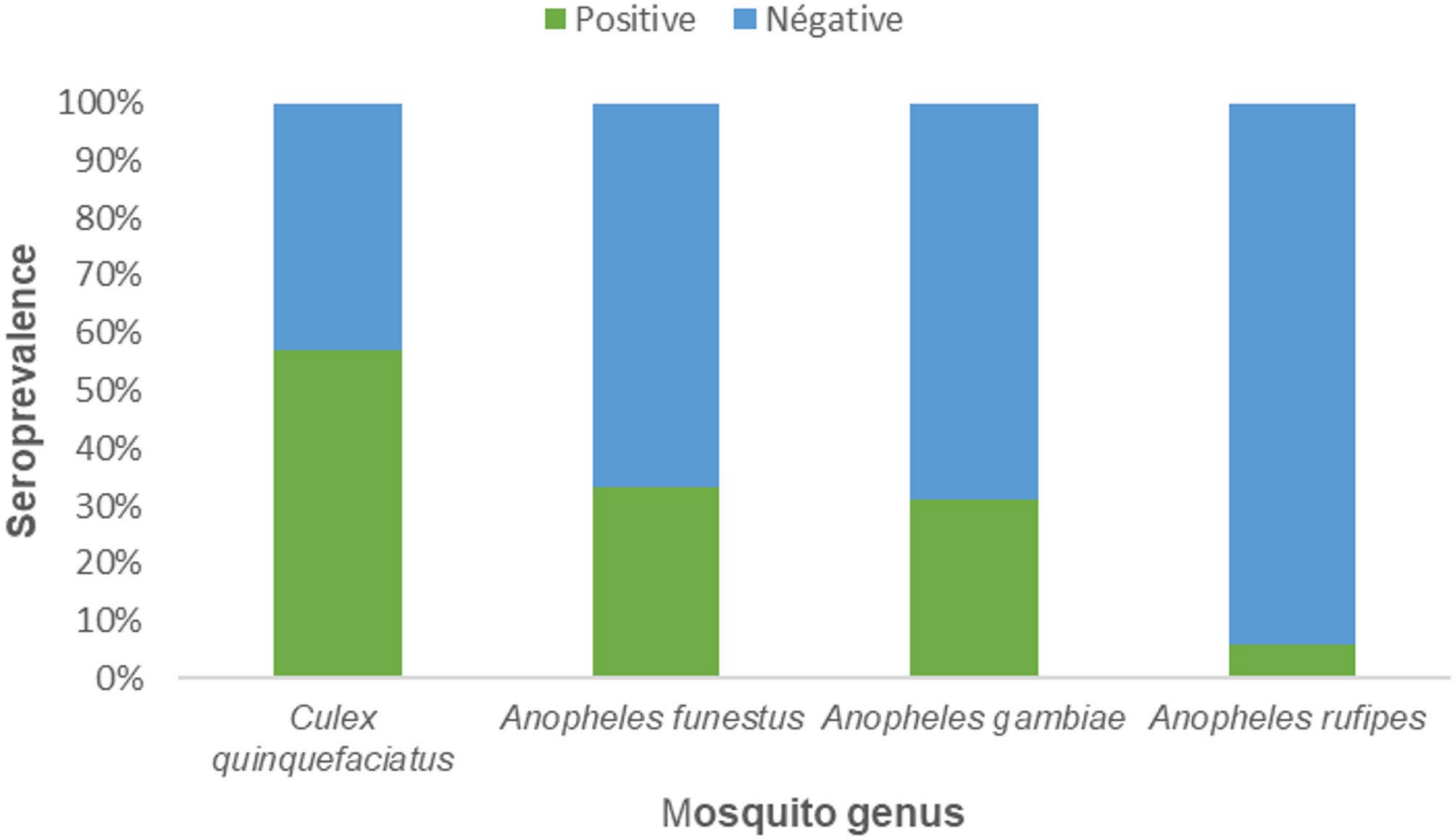
Mosquito-based SARS-CoV-2 seroprevalence by mosquito species.

To identify mosquito species best suited for xenosurveillance-based estimation of SARS-CoV-2 seroprevalence, we compared seropositivity rates among blood-fed mosquito species. *Culex quinquefasciatus* showed the highest proportion of seropositive blood meals (21/36, 58.3%), whereas *Anopheles rufipes* showed the lowest (1/34, 2.9%). *Anopheles gambiae* (141/462, 30.5%) and *Anopheles funestus* (52/158, 32.9%) showed intermediate seroprevalence levels (Fig. 3). seropositivity differed significantly across species (χ² = 25.33, *p* < 0.001).

## Discussion

This study provides evidence of SARS-CoV-2 circulation in rural areas of Burkina Faso during the COVID-19 pandemic using sampling based on naturally blood-fed mosquitoes, as an alternative to conventional human serological sampling. This approach offers a non-invasive means of population-level sero-monitoring in settings where access to healthcare and diagnostic services is limited.

The mosquito species composition observed in this study was dominated by Anopheles species, which is consistent with the rural setting of the sampling sites, where this genus is known to be highly prevalent. The predominance of Anopheles mosquitoes likely reflects both ecological factors specific to rural environments and the proximity of breeding and resting sites associated with human dwellings in villages. In addition, indoor collections performed in the early morning are particularly effective for sampling endophilic and nocturnal mosquitoes that rest indoors after blood feeding, a behavior characteristic of many Anopheles species.

Mosquito-based SARS-CoV-2 seroprevalence was 26.56% in areas near Ouagadougou and 35.14% near Bobo-Dioulasso in October and November 2021. These seroprevalence levels indicate substantial viral circulation in the human populations on which the mosquitoes fed and are aligned with the epidemiological context of that period. The detection of human anti–SARS-CoV-2 antibodies in mosquito blood meals reflects prior infection of human hosts and therefore serves as an indirect indicator of population exposure to SARS-CoV-2. For the same period, human seroprevalence reported by Struck et al. was 55.7% in Bobo-Dioulasso and 37.4% in Ouagadougou^20^. While mosquito-based seroprevalence values were systematically lower than those reported in human studies, this difference is expected given the indirect nature of xenosurveillance. These findings suggest that mosquito-based seroprevalence reflects broad spatial trends in human exposure rather than precise estimates of individual-level serostatus. The overall mosquito-based seroprevalence observed in this study (31.16%) is comparable to that reported by Krajacich et al. in Mali in early 2021 (25.1%)^17^, further supporting the validity of this approach across West African settings. Household-level seroprevalence estimates were higher than mosquito-based estimates but followed similar spatial patterns, with localities exhibiting higher mosquito seroprevalence also showing higher household positivity, except in Bombi. This difference is expected, as a household was classified as positive when at least one mosquito tested positive, whereas mosquito-based seroprevalence reflects positivity at the level of individual insects. Collecting multiple blood-fed mosquitoes per household therefore increases the likelihood of detecting at least one positive sample.

Previous studies have shown that antibodies can remain detectable in mosquito blood meals for up to 24 h following feeding^17^. Assuming that mosquitoes collected in this study fed during the preceding night, the estimated interval between blood feeding and collection (approximately 4 to 14 h) is unlikely to result in substantial antibody degradation. Consequently, the observed seropositivity likely reflects antibody levels present in human hosts at the time of blood feeding.

Comparison of seroprevalence across mosquito species revealed significant differences, suggesting that mosquito species may vary in their suitability for antibody detection through xenosurveillance. This pattern likely reflects species-specific ecological and biological characteristics, including feeding preferences, blood meal volume, and digestion dynamics. *Culex quinquefasciatus*, which exhibited the highest seroprevalence in this study, is a highly anthropophilic and endophilic species commonly found in close proximity to human dwellings^21^, which may partly justify its greater capacity to capture human antibodies during blood feeding.

Lack of data on the origin of the blood meal and the proportion of mosquitoes feeding on the same individuals are among major limitations of this study, and do not allow this seroprevalence to be extrapolated to the exact seroprevalence in the human population. However, these results provide clear evidence of SARS-CoV-2 circulation in rural communities where conventional surveillance is limited. Xenosurveillance using indoor-collected blood-fed mosquitoes represents a cost-effective, logistically simple, and ethically less constrained alternative to human blood sampling, particularly in resource-limited settings.

## Conclusions

In this study, we evaluated SARS-CoV-2 exposure rates in rural communities of Burkina Faso using the analysis of naturally blood-fed mosquitoes as a proxy for human serological sampling. The detection of human anti–SARS-CoV-2 antibodies in mosquito blood meals provides evidence of viral circulation in settings where conventional surveillance is limited. These results demonstrate the feasibility of mosquito-based xenosurveillance as a non-invasive and operational approach for estimating SARS-CoV-2 seroprevalence in remote communities. Beyond the scope of the present study, the integration of mosquito blood-meal analyses with blood source identification could, in future investigations, help monitor pathogen exposure in human populations and potentially provide insights into pathogen circulation at the human–environment interface within a broader One Health perspective.

## Supplementary Information

Below is the link to the electronic supplementary material.

Supplementary Material 1

## Data Availability

All data generated or analyzed during this study are included in this published article and its supplementary information files.

## Abbreviations

ELISA: Enzyme-Linked Immuno Assay
EID: Emerging infectious diseases
PBS: Phosphate buffered saline
